# Flip the Clinic: A Digital Health Approach to Youth Mental Health Service Delivery During the COVID-19 Pandemic and Beyond

**DOI:** 10.2196/24578

**Published:** 2020-12-15

**Authors:** Tracey A Davenport, Vanessa Wan Sze Cheng, Frank Iorfino, Blake Hamilton, Eva Castaldi, Amy Burton, Elizabeth M Scott, Ian B Hickie

**Affiliations:** 1 Brain and Mind Centre The University of Sydney Sydney Australia; 2 headspace Camperdown Sydney Australia

**Keywords:** health information technologies, clinical staging, youth, mental health, transdiagnostic, eHealth, routine outcome monitoring, adolescent, mental health services, health services, telemedicine, monitoring, outcome, young adult, COVID-19

## Abstract

The demand for mental health services is projected to rapidly increase as a direct and indirect result of the COVID-19 pandemic. Given that young people are disproportionately disadvantaged by mental illness and will face further challenges related to the COVID-19 pandemic, it is crucial to deliver appropriate mental health care to young people as early as possible. Integrating digital health solutions into mental health service delivery pathways has the potential to greatly increase efficiencies, enabling the provision of “right care, first time.” We propose an innovative digital health solution for demand management intended for use by primary youth mental health services, comprised of (1) a youth mental health model of care (ie, the Brain and Mind Centre Youth Model) and (2) a health information technology specifically designed to deliver this model of care (eg, the InnoWell Platform). We also propose an operational protocol of how this solution could be applied to primary youth mental health service delivery processes. By “flipping” the conventional service delivery models of majority in-clinic and minority web-delivered care to a model where web-delivered care is the default, this digital health solution offers a scalable way of delivering quality youth mental health care both in response to public health crises (such as the COVID-19 pandemic) and on an ongoing basis in the future.

## Introduction

The COVID-19 pandemic has created a major public health crisis that has led to multidisciplinary national coordination in which physical distancing and social isolation are being mandated to flatten predicted morbidity and mortality curves [1]. This has led to the emergence of a second public health crisis due to the onset or exacerbation of poor mental health symptoms caused by the social and economic impacts of the COVID-19 pandemic, leading to increased mental health service demand [2].

In response to the pandemic, the Australian health system has embraced existing telehealth solutions in which consumers receive care through telephone calls, video calls, or the internet. In March 2020, Medicare moved to support telehealth sessions through temporary Medicare Benefits Schedule (MBS) item numbers that include receiving care via information and communication technologies [3]. Importantly, this has provided a mechanism for many Australian mental health services to continue to operate “business as usual” even though they have been required to close their front doors.

The COVID-19 pandemic has necessitated the establishment and improvement of the technological infrastructure required to execute these temporary, stopgap solutions. Now that the groundwork has been laid, it is time to capitalize on these infrastructure developments and adapt our public mental health system to allow true digital health that fully integrates health information technologies (HITs) into service delivery models of care.

## The Need for Digital Health

Pre–COVID-19, the World Economic Forum highlighted massive problems in mental health service provision (eg, stigma, consumer-reported poor care experiences, late intervention, mental health treatment isolated from other physical and social needs, poor resource allocation, and service fragmentation [4]) and called for the “rapid deployment of smarter, digitally enhanced health services” as a means to address issues of demand [5]. More recently, the World Health Organization has highlighted the need to urgently increase mental health service capacity in response to the COVID-19 pandemic [6]. Physical distancing, social isolation, fear of contagion, and the sudden loss of family members are being further impacted by distress caused by loss of income and employment [6], with increased rates of stress, anxiety, depression, anger, and fear already being reported worldwide [7]. Within Australia, the Federal Treasury forecasts an unemployment peak of 10%-11%, which translates to an approximate jobless rate of 24% for Australian young people [8]. These trends are particularly concerning, as major mental disorders and substance use disorders affect at least one in four young people by 25 years of age and are associated with significant disability and premature death in this group worldwide [9]. The increase in youth unemployment as a result of the COVID-19 pandemic will further compound the vulnerability of young people as a group.

Already, many Australian mental health and suicide prevention services have reported an increase in demand during the COVID-19 pandemic [10], highlighting the need to increase service capacity to mount an effective response. Further, a recent dynamic simulation modelling study estimated that for young people, the impact of this secondary public health crisis will result in a 21% increase in mental health–related emergency department (ED) presentations, a 22% increase in self-harm hospitalizations, and a 23% increase in deaths by suicide [11]. Importantly, this model also found that increasing the provision of general practitioners, psychiatrists, and allied services by 11% per year and Community Mental Health Centre service capacity by 10% (resulting in a total youth mental health service capacity increase of 40%) alone would not have a major impact. However, if this capacity increase were combined with digital health (or technology-enabled care) and post–suicide attempt aftercare, this combination of strategies would result in an 8%-10% reduction in ED presentations, self-harm hospitalizations, and deaths by suicide [11].

A key priority should therefore be to immediately increase the speed and quality of the responses of the mental health system to increases in service demand. Previous research has established the utility of “blended” approaches to mental health care that combine face-to-face treatment with web-based tools such as internet-delivered mental health interventions and self-monitoring apps [12,13]. Redesigning youth mental health service delivery pathways to integrate digital health solutions would build on the progress that has been made in the past (as well as our initial response to the COVID-19 pandemic) and establish a mental health workforce that is equipped to respond to surges in demand, both now and on an ongoing basis in the future [2].

## Digital Health as a Demand Management Strategy

In academic literature, digital health has been defined as the “cultural transformation of how disruptive technologies that provide digital and objective data accessible to both caregivers and patients lead to an equal level physician-patient relationship with shared decision-making and the democratization of care” [14]. In clinical practice, our research team has more recently defined digital health as the “provision of guided mental health care where consumers navigate a rapid and more effective system experience of service entry, skilled assessment, and multidisciplinary and coordinated care, as well as ongoing outcome-based monitoring.” Importantly, the latter definition situates consumers more strongly within the service operational processes through which mental health care is delivered, and it places additional focus on system-level factors such as demand management.

Over the past decade, the mental health sector has experienced a substantial surge in the development of numerous apps and technologies for mental health [15,16]. On an individual level, web-based cognitive behavior therapy modules and web-based programs have been found to lower anxiety and depression symptoms in young people [17]; also, many studies have found that young people are receptive to accessing HITs for their mental health in conjunction with face-to-face care [18-20]. Providing these HITs as an adjunct resource to support young people in early stages of illness could promote agency instead of reliance on an overburdened mental health care system, and it would be particularly helpful for young people who experience barriers to regularly accessing in-clinic care, such as geographic remoteness [21].

The Australian mental health system currently funds a limited number of psychologist appointments per year for each individual consumer, and senior clinicians in conventional mental health services spend extensive time conducting thorough face-to-face assessments [22]. The successful integration of HITs to support this process could represent significant time-saving and increase efficiencies in assessment, care coordination, and monitoring; thus, the time of senior clinicians could be reallocated to delivering skilled interventions [23,24]. On the service level, HITs have already demonstrated potential for enabling an appropriate and timely response for young people reporting higher levels of suicidality [25], facilitating broader assessment of the totality of a young person’s needs, and enabling senior clinicians to move away from traditional evaluations toward detailed data-driven assessments [23].

It is now time to take the next step of integrating HITs into mental health service delivery models of care in a way that meets increasing consumer demand and expectations, maximizes use of senior clinician skill, enables truly person-centered and collaborative care, and preserves service quality [26,27]. Although it remains important to develop and assess the efficacy of web-based interventions, an equally important task is to determine how to integrate and use these new technologies effectively [28]. Digitizing mental health service delivery, or “flipping” conventional models of service delivery so that a larger proportion of care is delivered via HIT-enabled solutions, would therefore address many barriers to help-seeking and service access, and take advantage of many of the benefits listed above.

The effectiveness of this approach would vary across mental health services; the optimal “flip” of in-clinic versus web-based service delivery is likely to be different for each service. We theorize that optimal level of “flip” will depend on the following factors: intensity of care provided by a mental health service (ie, primary and specialist services); digital familiarity of service staff and consumers; continuity of the service’s workforce; the aggregate physician-patient relationship; level of communication for change management; and local technology infrastructure and internet connectivity. It is likely that each service will need to iteratively identify its optimum level of “flip” over time. Regardless, the basic principles of using HITs to enhance key service operational processes and improve the quality of mental health care would apply.

## Presenting an Innovative Digital Health Solution

Our research team has recently developed an innovative digital health solution that comprises two components: (1) a measurement-based (data-driven) model of highly personalized youth mental health care, the Brain and Mind Centre (BMC) Youth Model; and (2) a dedicated HIT designed to deliver this model of care (exemplified by, but not limited to, the InnoWell Platform).

The BMC Youth Model [29] recognizes that while it is crucial to intervene at early stages of mental illness (ie, early intervention), these early stages also tend to be characterized by nonspecific symptoms that overlap disease categories and do not meet diagnostic criteria. Under the BMC Youth Model, a young person’s needs are assessed on a multidimensional outcomes framework, and their underlying pathophysiological mechanisms and illness trajectories are considered. They are also allocated to a clinical stage that represents their relative state of illness severity and persistence, with Stage 1a representing nonspecific symptoms, Stage 1b representing attenuated syndromes, and Stage 2+ indicating more discrete and persistent disorders [30]. The BMC Youth Model integrates over 10 years of neurobiological, neuropsychological, and clinical research into a single framework of youth mental health care, and it was designed to be supported by two mechanisms: education and training for service staff (Scott et al, in submission) and a dedicated HIT whose key functionalities would be specially developed to support the core concepts of the BMC Youth Model. We developed the InnoWell Platform in our work in Australia for this purpose.

## Details of the InnoWell Platform

The InnoWell Platform is an industrial-grade HIT manufactured by InnoWell Pty Ltd (a joint venture between The University of Sydney and PricewaterhouseCoopers [PwC] Australia). This platform is listed in the Australian Register of Therapeutic Goods (software as a medical device, class 1, ARTG ID 315030) as a “*customisable digital toolkit to assist assessment, monitoring, and management of mental ill health and maintenance of wellbeing. It does this by collecting, storing, scoring, and reporting personal and health information back to consumers and their health professionals to promote collaborative care partnerships*” [31]. Since its inception (wireframe and prototype stage), the InnoWell Platform has been continually co-designed with target end users, including consumers and their supportive others, health professionals, and other service staff [24].

Importantly, the BMC Youth Model is not wedded to one HIT; it can be supported by any HIT that has been designed to provide highly personalized and measurement-based care according to its key clinical and scientific principles. The development of this dedicated HIT would ideally involve a co-design process with target end users (consumers and their supportive others, clinicians, and other service staff). Co-design is the deliberate, considered, and nontokenistic involvement of target end users in the design and development of a technology. In addition to boosting the voices of marginalized groups, co-design increases the acceptability of the end product [32]. Because its primary purpose is to support the delivery of the BMC Youth Model, the dedicated HIT would not provide diagnoses and medical advice; instead, it would support the provision of diagnoses and medical advice based on the specifications of the mental health services in which it is implemented. In other words, each service can customize the care options recommended by the dedicated HIT and the conditions under which these care options are recommended.

## Operating a “Flipped Clinic”

Although the predicted surge in demand following the COVID-19 pandemic will exacerbate existing difficulties faced by young people in accessing quality mental health care and by service providers in delivering said care, this can be mitigated with HIT-enabled demand management strategies. In conventional service delivery models, in-clinic care tends to be emphasized and provided regardless of symptom severity and persistence, with web-based service delivery modalities (eg, voice or video calling) offered as “backup.” A redesigned service delivery model that “flips” to a default of web-based service delivery, and the integration of HITs into service delivery pathways to take advantage of their enhanced assessment, care coordination, and monitoring capabilities, could result in quicker waiting times (or even no more waitlists) and more efficient resource allocation. Offering young people at earlier and milder stages of illness (ie, Stage 1a) the opportunity to access psychoeducation and web-based interventions with the support of a junior clinician would free up service resources (including senior clinician time), enabling them to be reallocated toward delivering higher-intensity interventions to young people at more severe stages of illness.

**Figure 1:**
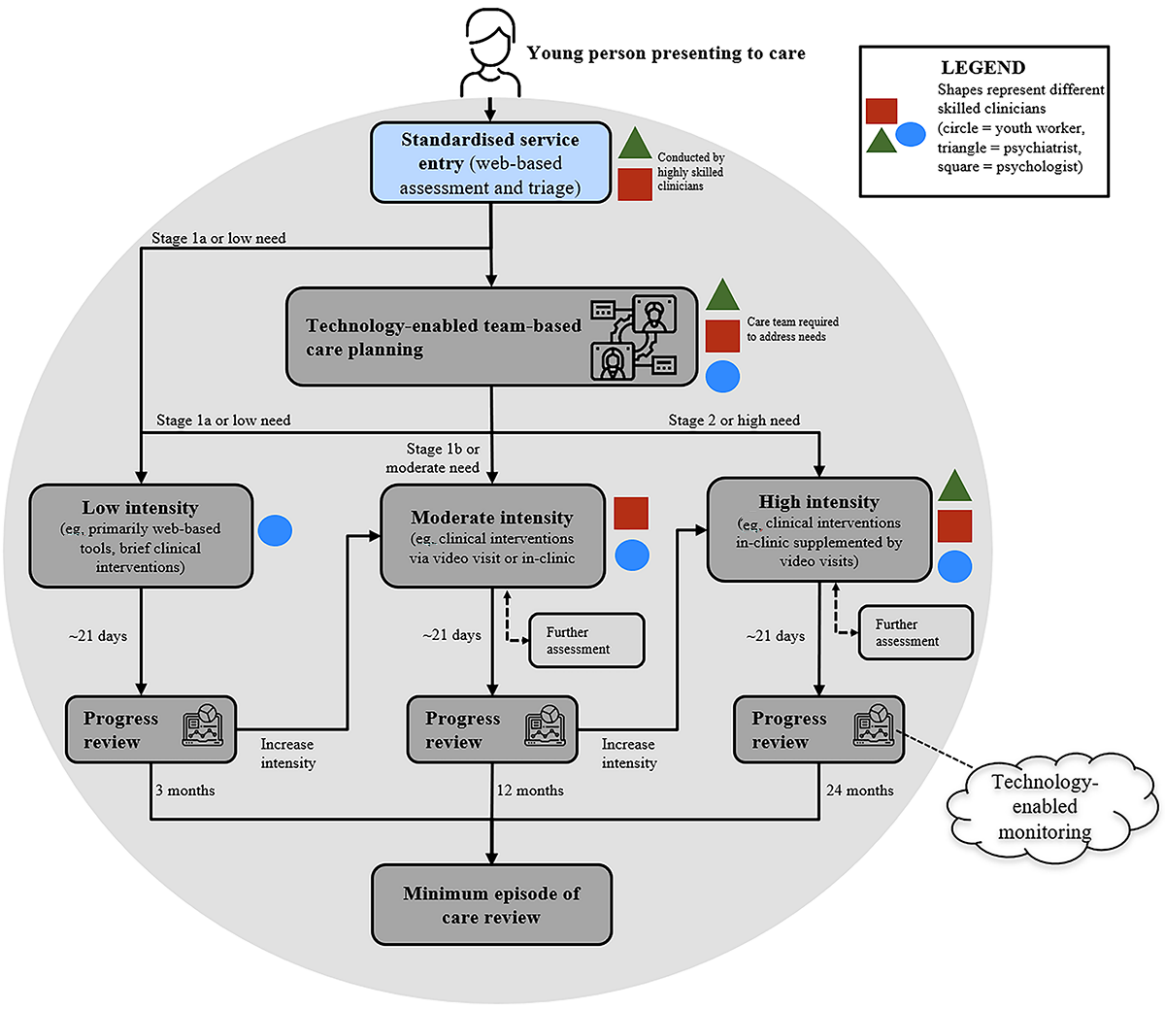
Flowchart demonstrating the implementation of our digital health solution within a primary youth mental health service.

Figure 1 shows a redesigned HIT-enabled mental health service model for primary youth (aged 12 to 25 years) that adopts our digital health solution. First, standardized service entry uses web-based assessment, triage, and sophisticated suicide escalation protocols [25] to help immediately determine best care pathways based on the young person’s risk and clinical stage. Care decisions are driven by the young person’s needs and focus on getting the right team involved as well as on identifying the type and length of care required, modes of delivery, and need for further assessment. Routine outcome monitoring also enables the care team to respond to the young person’s changing needs (eg, increasing the intensity of care or changing the care team); this outcome monitoring can occur at a micro (daily or weekly) or macro (monthly or yearly) level.

Table 1 provides further operational details on how each service process could be digitally “flipped” with specific reference to the dedicated HIT, and it outlines the minimum functionalities the HIT should possess. In addition to detailing the intake, assessment, triage, care coordination, tracking, and monitoring processes depicted in Figure 1, Table 1 adds an additional step for service quality improvement, whereby key performance data can be regularly reviewed by service management who can then enact changes to service delivery to improve the quality of mental health care.

**Table 1:** Operational protocol demonstrating how to “flip” a primary youth mental health service using our digital health solution.

Stage	Actions
Intake	The young person or their supportive other contacts the service (eg, telephone, web, email, walk-in). The service conducts an intake screen and invites them to use the digital health solution (via the dedicated HIT). The young person accepts the invite and sets up an account on the dedicated HIT.
Assess	The young person completes a web-based multidimensional assessment that covers key mental health domains as well as social and occupational function, physical health, and substance misuse (20-40 minutes), ideally within 72 hours. The young person invites their supportive others to also contribute data through a shorter “summary” assessment (5 minutes).
Triage	Triage is conducted by a senior clinician, such as a psychiatrist (and registrar), clinical psychologist, or mental health nurse. Triage is determined by real-time “escalations” that trigger upon detection of clinical risk (eg, meeting a certain threshold for suicidal thoughts/behaviors or abnormal mental states such as mania and psychosis), clinical staging, and current level of need. Triage is completed the next business day after a young person has finished the web-based multidimensional assessment. Urgent cases (suicidal thoughts/behaviors, mania, psychosis) are prioritized to be seen immediately using video-visit functionality [23], of which a small proportion are referred straight to acute care services.
Care	Ongoing care pathways are matched to the appropriate type, intensity, and duration of intervention [29]. Using a multidisciplinary care team approach, these critical decisions are made early in the care pathway by senior clinicians to ensure accurate and efficient allocation of young people to care. This represents a secondary “flip” in the clinic, whereby senior clinicians are involved earlier in the care pathway as opposed to later. Stage 1a cases are directed to use web-based care tools for a minimum of three months in association with a junior clinician. Because Stage 1b cases are at greater risk of transitioning to more severe stages of illness compared to Stage 1a cases [33], these should be reviewed with video-visit functionality [23] and directed to use care options in partnership with their multidisciplinary care team for a minimum of 12 months. Stage 2+ cases receive more specialist care in face-to-face settings for a minimum of two years.
Track	Active tracking of symptoms/functioning by encouraging young people (and invited supportive others) to complete a “check-in assessment” at least every 21 days. Innovative use of the dedicated HIT should be considered, wherein a service could use the video-visit functionality to track young people in real time (eg, up to three 10-minute sessions per week).
Monitor	In conventional primary youth mental health services, only 20%-30% of cases show reliable improvement; 10%-25% of cases will deteriorate significantly over approximately six months; and, the majority of cases are left with persistent distress and/or impairment (i.e. no change) [22]. Therefore, the dedicated HIT should be used for real-time review of deteriorating or non-changing cases through routine outcome monitoring that encourages care plans to change in response to outcome data, such as changing the type, intensity, and duration of intervention.
Review	As part of a service’s “quality improvement cycle,” management could review service-level data collected by the dedicated HIT during routine care to evaluate (overall and by clinician) the clinical safety; accessibility and equity; effectiveness and outcomes; acceptability and satisfaction; efficiency, expenditure and cost; appropriateness; continuity and coordination; and workforce competence and capability [24].

## Further Considerations for a “Flipped Clinic”

Our proposed “flipped clinic” service delivery model was developed following many years of clinical and co-design research with participants in the Australian mental health system, including consumers and their supportive others, health professionals, and service providers, to address major problems faced by users of this system: a limited supply of mental health professionals who are burdened with administration and assessment [22]; a lack of communication between mental health providers [4,18]; and the “tyranny of distance” [18,21]. This model proposes an innovative redesign of mental health service delivery that harnesses the capabilities of digital technologies (including, but not limited to, eHealth or mobile health [mHealth] interventions and other HITs) to enhance the provision of care for all participants in the system. However, some key considerations remain for those wishing to adopt this model or to enact similar widespread digital health system reforms.

As this model was developed using the Australian health system (a predominantly fee-for-service system) as a reference, it is likely to be generally applicable to similar systems. However, further work would still be required to adapt the model to a different country (with differences in service delivery mechanisms as well as facilitators and barriers to mental health care delivery) or funding model (eg, blended or pay-for-performance approaches). Within our digital solution, the “quality improvement cycle” (Table 1) could be particularly relevant for payment systems that are not primarily fee-for-service, and it could be further developed.

Our digital solution was also developed primarily from research activities at and around a primary youth mental health service aimed at providing care to Australian young people aged 12-25 years. The developmental heterogeneity of this age group has been widely researched [34,35]; as such, any solution for this age group would have to cater to a mixture of children (aged <15 years), adolescents (aged 15-18 years), and transition-aged youth (aged 18-25 years) with a diversity of life experiences, clinical presentations, and clinical trajectories. Because our research team was working with a mental health service that specialized in treating youth, we were able to integrate our digital solution into existing service protocols for youth of different ages. However, other services or systems looking to adopt our solution may be required to adapt our approach, for example by developing different versions of our protocol for different youth age groups, and involving supportive others more heavily for young people below a certain age (with this age varying across jurisdictions and cultures).

Finally, although our digital solution was developed to address inequalities in access to youth mental health care in Australia, such as the “tyranny of distance” faced by young people in rural and remote areas [18,21], it does not yet address other existing inequalities, such as English literacy and different levels of access to digital technologies or the internet. A mixture of solutions will be required to address these inequalities, such as translating the dedicated HIT to different languages or designing offline functionality. However, these inequalities are also inherent to the broader Australian mental health system [36], and widespread government action will be required to address them (such as through infrastructure upgrades to improve nationwide internet connectivity).

## Conclusion

Our digital health solution of digitally “flipping” clinics is a promising new demand management strategy for primary youth mental health services that aims to provide quality mental health care by leading consumers through a rapid experience of service entry, comprehensive assessment, multidisciplinary care, and routine outcome-based monitoring. Adopting this solution and establishing the required technological infrastructure could increase efficiencies in accessing and delivering quality mental health care and could also enable a dynamic response to public health crises such as the COVID-19 pandemic.

## Abbreviations

BMC: Brain and Mind Centre
ED: emergency department
HIT: health information technology
MBS: Medicare Benefits Schedule
mHealth: mobile health
PwC: PricewaterhouseCoopers
